# Subclinical structural atypicality of retinal thickness and its association with gray matter volume in the visual cortex of maltreated children

**DOI:** 10.1038/s41598-024-62392-6

**Published:** 2024-05-20

**Authors:** Akiko Yao, Shota Nishitani, Yutaka Yamada, Hideyuki Oshima, Yuka Sugihara, Kai Makita, Shinichiro Takiguchi, Natasha Y. S. Kawata, Takashi X. Fujisawa, Hidehiko Okazawa, Masaru Inatani, Akemi Tomoda

**Affiliations:** 1 Research Center for Child Mental Development, University of Fukui, 23-3 Matsuoka-Shimoaizuki, Eiheiji-cho, Fukui, 910-1193 Japan; 2 Division of Developmental Higher Brain Functions, United Graduate School of Child Development, Osaka University, Kanazawa University, Hamamatsu University School of Medicine, Chiba University, and University of Fukui, Osaka, Japan; 3 Life Science Innovation Center, School of Medical Sciences, University of Fukui, Fukui, Japan; 4 Department of Ophthalmology, University of Fukui, Fukui, Japan; 5 Department of Child and Adolescent Psychological Medicine, University of Fukui Hospital, Fukui, Japan; 6 Biomedical Imaging Research Center, University of Fukui, Fukui, Japan

**Keywords:** Paediatric neurological disorders, Human behaviour, Trauma

## Abstract

Childhood maltreatment is reportedly associated with atypical gray matter structures in the primary visual cortex (V1). This study explores the hypothesis that retinal structures, the sensory organs of vision, are associated with brain atypicality and child maltreatment and examines their interrelation. General ophthalmologic examinations, visual cognitive tasks, retinal imaging, and structural magnetic resonance imaging (MRI) were conducted in children and adolescents aged 9–18 years with maltreatment experiences (CM) and typically developing (TD) children. The retinal nerve fiber layer (RNFL), the most superficial of the ten distinct retinal layers, was found to be significantly thinner in both eyes in CM. While whole-brain analysis using Voxel-based morphometry revealed a significantly larger gray matter volume (GMV) in the thalamus in CM, no significant correlation with RNFL thickness was observed. However, based on region-of-interest analysis, a thinner RNFL was associated with a larger GMV in the right V1. Although it cannot be ruled out that this outcome resulted from maltreatment alone, CM demonstrated subclinical structural atypicality in the retina, which may also correlate with the immaturity of V1 development. Examination of retinal thickness offers a novel clinical approach to capturing characteristics associated with childhood maltreatment.

## Introduction

Childhood maltreatment is a serious social risk factor that leads to atypical brain development, subsequently contributing to an increased prevalence of psychiatric disorders, including personality disorders, and increased suicide rates^1,2^. Despite various preventive efforts by the World Health Organization (WHO) and many nations—ranging from epidemiological studies and social awareness campaigns to changes in laws^3^—biological perspectives on early detection efforts remain limited. Several studies have explored potential biomarkers for the early detection of childhood maltreatment, including brain MRI^4–8^, epigenomic analyses^9–11^, and hormone and cytokine^12–14^ measurements. However, these investigations are largely pilot studies, lacking established indicators and remaining in the early stages of exploring a wide range of possible biomarkers that reflect childhood maltreatment features.

In studies involving young adults with a history of childhood sexual abuse or witnessed domestic violence, or children diagnosed with reactive attachment disorder (RAD) and exposed to various types of maltreatment, a common finding is a reduced gray matter volume (GMV) in the left primary visual cortex (V1), as reported by Voxel-based morphometry (VBM) brain MRI^4,6,15^. This atypicality in V1 has been interpreted as an influence on the vulnerability of the network responsible for emotion regulation. Reduced cortical thickness in the frontal and temporal cortices and parahippocampal gyrus, considered important for emotion regulation in adolescents aged 13–20 years who experienced physical or sexual abuse, supports this interpretation^16^. While there is scattered evidence on the effects of childhood maltreatment on brain regions controlling other primary sensory cortices^17^, no studies have explored atypicality in the respective sensory organs connected to these brain regions.

Visual information is transmitted from the retina through the optic nerve, optic chiasm, and lateral geniculate nucleus in the thalamus to reach V1. Optical coherence tomography (OCT) has become a common noninvasive imaging technique for examining retinal layer structures, primarily employed in ophthalmic diseases^18^. Recent attempts have been made to examine whether neurodegenerative diseases can be indirectly inspected by monitoring the retinal condition. The retina, as part of the nervous system, is composed of neurons^19^. Most of these studies are currently focused on dementia, Alzheimer's disease^20,21^, Parkinson's disease^22^, and multiple sclerosis^23^. However, beyond neurodegenerative diseases, recent studies have indicated that retinal layer thickness exhibits atypical structures in some psychiatric and neurodevelopmental disorders, including schizophrenia^24,25^, bipolar disorder^26^, major depressive disorder^27–29^, and autism spectrum disorder (ASD)^30^. More recently, a study revealed a decrease in the number of retinal ganglion cells in a mouse model of childhood neglect exposure^31^. Despite these attempts, no studies have directly examined the non-invasive imaging of retinal layer structures using OCT in relation to childhood maltreatment experiences.

This study aims to test the hypothesis that childhood maltreatment leads to retinal structural atypicality, reflecting structural atypicality in the brain. Additionally, to date, no studies have focused on visual sensory organ structures and functions in children and adolescents with maltreatment experiences (CM). To achieve this goal, a case–control study was conducted on CM and age- and sex-matched typically developing (TD) children. The study included comprehensive examinations for vision, including retinal imaging with OCT, general ophthalmologic examinations, color tests, and visual cognitive tasks. Previous studies examining retinal structures in neurological and psychiatric disorders have only analyzed their relationship with brain structures and did not directly examine their association with each other. Therefore, the secondary aim of this study is to investigate whether there is a direct relationship between brain structure and retinal thickness, in addition to characterizing the brain structure of CM compared to that of TD children by simultaneously examining brain MRI (Table 1).

**Table 1:** Demographics and clinical characteristics of participants.

	CM (*n* = 21)		*n*	TD (*n* = 23)		*χ*^2^	*t*	*P*
	M	SD		M	SD	*n*		
Male participants			12			11	0.10	0.75
Age (y)	14.0	(2.7)		14.3	(2.6)		−0.45	0.66
Right-handedness			18			22	0.38	0.53
Types of maltreatment								
Physical abuse			9			–		
Emotional abuse			10			–		
Neglect			17			–		
Sexual abuse			1			–		
Number of types of maltreatment	1.7	(0.8)				–		
Duration (years) of maltreatment^a^	6.2	(3.5)				–		
Duration (years) elapsed from maltreatment^b^	6.7	(3.5)				–		
WISC-IV FSIQ	92.1	(8.6)		105.4	(12.0)		−4.17	< 0.001****
DSRS-C	13.2	(7.3)		8.4	(5.6)		2.45	0.02*
SDQ total	11.2	(4.8)		5.6	(4.5)		3.98	< 0.001****

WISC-IV; Wechsler Intelligence Scale for Children ver. IV, FSIQ; Full-Scale I.Q., DSRS-C; Depression Self-Rating Scale for Children, SDQ; Strength and Difficulties Questionnaire.

^a^Three CM children had no precise record. ^b^ One CM child had no precise record.

*P < 0.05; ****P < 0.001.

## Results

### Ophthalmologic examinations results

No clear clinical visual abnormalities were observed in any of the children, except for two in the TD group who did not pass the Ishihara color blindness test. As a result, another color test assessing subtle color discrimination for these two was excluded from the analysis. However, data from other measurements were utilized. As shown in Table 2, no significant group differences were found in general ophthalmological examinations, except for left lens thickness (LT) (*P* < 0.05).

**Table 2:** Ophthalmologic examinations.

	OD (Right)				OS (Left)			
	CM (*n* = 21)	TD (*n* = 23)	*t*	*P*	CM (*n* = 21)	TD (*n* = 23)	*t*	*P*
IOP (mmHg)	14.6 ± 0.4	14.9 ± 0.5	−0.48	0.63	13.6 ± 0.5	14.7 ± 0.7	−1.42	0.16
Kerato								
R1 (mm)	7.9 ± 0.1	8.0 ± 0.1	−1.14	0.26	7.9 ± 0.1	8.0 ± 0.1	−1.32	0.19
R2 (mm)	7.7 ± 0.1	7.8 ± 0.1	−0.58	0.57	7.7 ± 0.1	7.8 ± 0.1	−0.77	0.45
R1_D	42.9 ± 0.4	42.4 ± 0.3	1.16	0.25	43.0 ± 0.3	42.4 ± 0.3	1.35	0.18
R2_D	43.9 ± 0.4	43.6 ± 0.3	0.69	0.50	44.0 ± 0.4	43.5 ± 0.4	0.78	0.44
R1_degree	136.3 ± 14.3	165.2 ± 7.3	−1.80	0.08	94.9 ± 18.2	75.3 ± 17.2	0.78	0.44
R2_degree	80.6 ± 2.6	83.0 ± 1.3	−0.85	0.40	90.6 ± 2.5	94.9 ± 2.1	−1.29	0.21
CYL_D	−1.0 ± 0.1	−1.2 ± 0.2	1.05	0.30	−1.0 ± 0.1	−1.2 ± 0.1	1.12	0.27
UCVA	0.5 ± 0.1	0.4 ± 0.1	0.57	0.57	0.5 ± 0.1	0.4 ± 0.1	0.75	0.46
CVA	1.0 ± 0.0	1.1 ± 0.0	−1.30	0.21	1.0 ± 0.0	1.1 ± 0.0	−1.30	0.20
SPH	−1.8 ± 0.7	−3.2 ± 0.4	1.50	0.14	−2.1 ± 0.6	−2.8 ± 0.5	0.74	0.47
AL (mm)	24.0 ± 0.4	24.7 ± 0.2	−1.24	0.23	24.0 ± 0.4	24.7 ± 0.3	−1.30	0.20
ACD (mm)	3.6 ± 0.1	3.7 ± 0.0	−0.63	0.54	3.6 ± 0.1	3.7 ± 0.0	−0.92	0.36
LT (mm)	3.4 ± 0.0	3.3 ± 0.0	2.00	0.06	3.5 ± 0.0	3.3 ± 0.0	2.04	0.05*
CCT (μm)	553.3 ± 4.3	556.0 ± 6.0	−0.37	0.72	553.1 ± 4.4	556.7 ± 5.6	−0.50	0.62
CFF (Hz)	45.6 ± 0.6	45.8 ± 1.0	−0.17	0.87	45.5 ± 0.7	45.7 ± 0.9	−0.16	0.87

Data are shown as mean ± standard error, IOP; intraocular pressure, D; diopter, CYL; Cylinder, UCVA; uncorrected visual acuity, CVA; corrected visual acuity, SPH; sphere, AL; axial length, ACD; anterior chamber depth, LT; lens thickness, CCT; central corneal thickness, CFF; critical fusion frequency.

*P < 0.05.

### Visual Activities Questionnaire (VAQ) results^32^

CM scored significantly better than TD for perceived visual function during ordinary activities (Whelch *t*-test: all eight subscales and the total score; multiple regression analysis adjusting for age and FSIQ: six subscales and the total score) (*Ps* < 0.05) (Supplementary Table S1).

### Farnsworth-Munsell 100-hue Test (FMT100) results^33^

CM exhibited significantly higher error scores for Tritanopes (Whelch *t*-test; *P* = 0.02, multiple regression analysis; *P* = 0.04) (Supplementary Table S2). No other significant group differences were observed.

### Optical coherence tomography (OCT), and OCT angiography (OCTA) results

As presented in Table 3, the RNFL for both eyes was significantly thinner in CM than in TD (*Ps* < 0.05, 9.0% (Right) and 9.2% (Left) thinner). As cognitive function has been reported to be correlated with retinal thickness^34,35^, additional analyses adjusting for age and FSIQ were conducted; however, this significance remained for RNFL (*Ps* < 0.05). No other significant segment differences were observed in the OCT images. No significant correlations were found within the CM group between RNFL thickness and potential indices of maltreatment severity, such as duration under the maltreatment situation (Right; *P* = 0.94, Left; *P* = 0.98) and the number of maltreatment types (Right; *P* = 0.92, Left; *P* = 0.74). In the OCTA, one TD child’s data could not be acquired because of a technical error. No significant group differences in microvasculature were observed in the OCTA images (Supplementary Table S3).

**Table 3:** Optical coherence tomography (OCT).

	CM (*n* = 21)	TD (*n* = 23)	Welch *t*-test		Multiple regression	
			*t*	*P*	*t*	*P*
OD (Right)						
RNFL	38.6 ± 0.9	42.7 ± 0.9	−3.22	0.002***	−2.49	0.02*
GCL+	63.6 ± 1.0	63.7 ± 0.9	−0.02	0.98	−0.36	0.72
GCL++	102.3 ± 1.5	106.4 ± 1.4	−2.01	0.05	−1.63	0.11
Choroid^a^	239.2 ± 13.3	228.2 ± 13.4	0.58	0.56	0.57	0.57
Retina	268.8 ± 3.9	269.3 ± 2.5	−0.10	0.92	−0.07	0.94
OS (Left)						
RNFL	39.0 ± 0.8	42.4 ± 0.8	−3.11	0.003***	−2.10	0.04*
GCL+	63.5 ± 1.0	63.8 ± 0.9	−0.16	0.88	−0.40	0.71
GCL++	102.5 ± 1.4	106.2 ± 1.2	−1.96	0.06	−1.43	0.16
Choroid^a^	235.0 ± 14.0	227.6 ± 12.8	0.40	0.69	0.28	0.78
Retina	270.1 ± 3.2	268.7 ± 2.5	0.30	0.77	0.47	0.64

Data are shown as mean ± standard error. Multiple regression analysis was adjusted with age and full-scale IQ. RNFL; retinal nerve fiber layer, GCL+; ganglion cell layer with inner plexiform layer, GCL++ (RNFL and GCL+), Choroid; from brunch membrane to choroid sclera junction, and Retina; from internal limiting membrane to outer segment/retinal pigment layer.

^a^One TD child had no data due to a technical error.

*P < 0.05; ***P < 0.005.

### Visual cognitive tasks results

As reflected in Supplementary Table S4, CM exhibited significantly longer reaction times (RT) for the target condition in “Visual search” (*P* = 0.004), for local and non-target conditions in the “Navon task” (*P* = 0.01, and *P* = 0.01, respectively), and a higher error rate for the “mental rotation task” (*P* = 0.03). No significant group differences were observed under the other conditions. These trends diminished when adjusting for age and FSIQ in the multiple regression analysis. The results indicated no significant group differences in the visual cognitive tasks under any task condition.

### Whole-brain VBM and the associations with retinal thickness results

**Figure 1:**
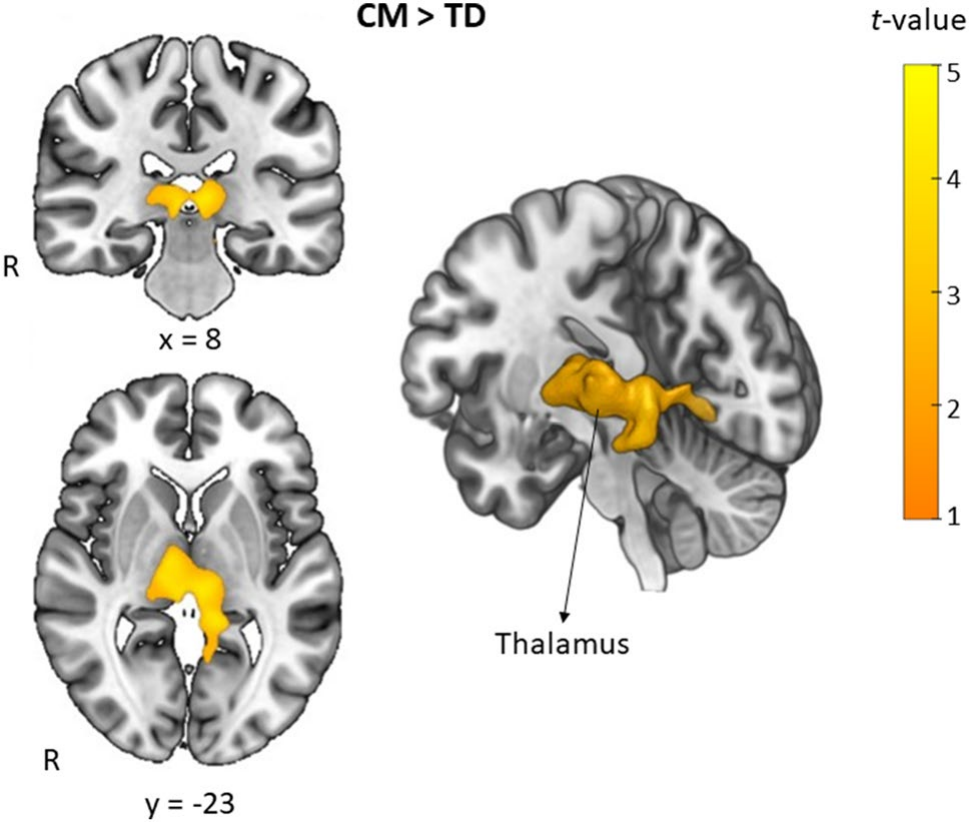
Structural differences in regional GMV between CM and TD children by whole-brain VBM. Significantly larger voxels (*P* = 0.001, FWE-corrected cluster level) found in CM compared to TD are depicted in yellow, identified as the thalamus (x = 8, y = −23, z = 4). The color scale represents *t*-values.

A significantly larger GMV cluster in the bilateral thalamus (MNI coordinates: x = −8, y = 27, z = 6; cluster size = 1229 voxels; *T* = 4.57; *P* < 0.001, family-wise error (FWE) corrected cluster level) was observed in CM compared to TD (Fig. 1). No other significant areas were identified using corrected cluster probability values. The β value from the identified area in the thalamus was extracted and used for correlation analyses of RNFL thickness in those segments that significantly differed between the groups. However, no significant association was found between the thalamus β value and RNFL thickness.

### Region-of-interest (ROI) analysis and associations with retinal thickness results

**Figure 2:**
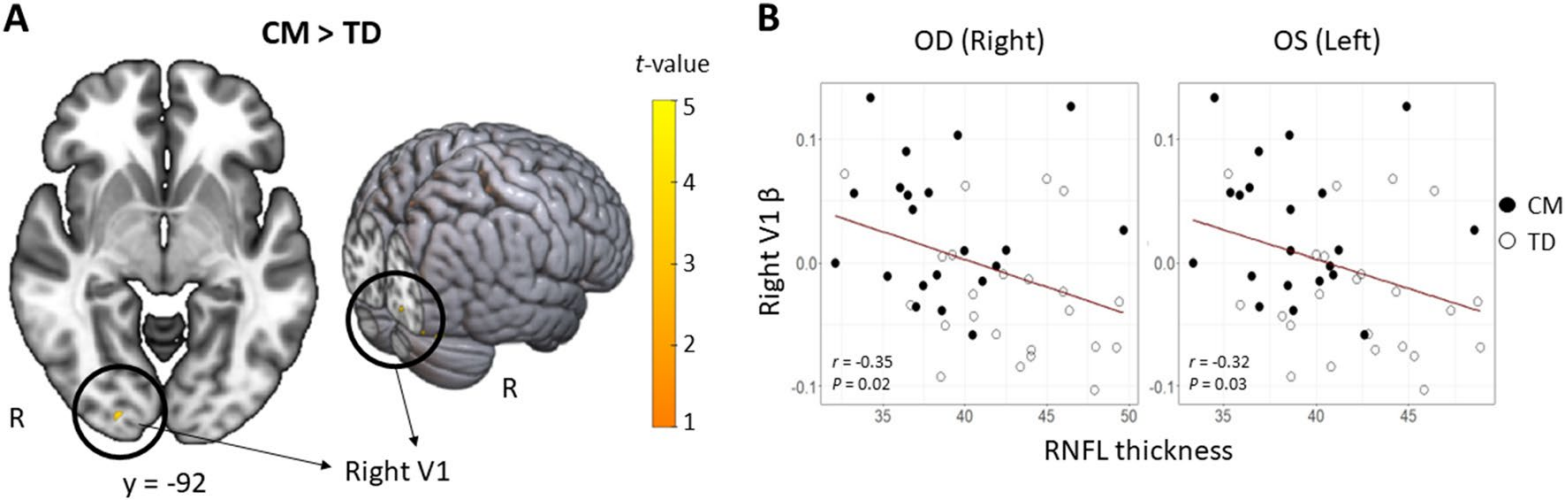
Structural differences in the GMV of right V1 between CM and TD children by ROI analysis and the association with the retinal thickness. (**A**) Significantly larger voxels (*P* < 0.001, FWE-corrected peak level) found in CM compared to TD are depicted in yellow (x = 20, y = −92, z = −6). (**B**) The scatter plots and regression lines (red) show the association between RNFL thickness and GMV of the right V1.

The GMV of the right V1 was larger (MNI coordinates: x = 20, y = −92, z = −6; *T* = 4.22; *P* = 0.020, FWE corrected peak level) in CM compared to TD (Fig. 2A). No other significant occipital areas with corrected peak probability values were identified. Across all participants, the β value of the right V1 was significantly correlated with both right (*r* = −0.35, *P* = 0.02) and left (*r* = −0.32, *P* = 0.03) RNFL thickness (Fig. 2B).

## Discussion

The primary objective of this study was to examine whether CM leads to retinal structural atypicality, which is analogous to structural atypicality in the brain. The results revealed that the macular RNFL in CM was thinner than that in TD. In the general ophthalmological examination, no ophthalmic disease or other pathological abnormality was detected in CM. In this study, cycloplegic was not used due to its invasiveness, but general ophthalmologic examinations, such as objective refraction, were conducted. However, no noticeable group differences were observed in these indices, and objective refraction in children is strongly influenced by accommodation; thus, it might not accurately account for refraction error evaluation. The measurement error increases with axial length (AL). However, the absence of group differences in AL (ranging from 24 to 25 mm, with participants with high myopia were not included) also suggests that refractive error did not influence the differences in retinal thickness. Retinal thickness was measured with the retinal magnification automatically adjusted according to the objective refraction, corneal curvature, and AL. These results suggest that the thinning of the macular RNFL results could not have been explained by refractive error, retinal magnifications, or other indices measured in the general ophthalmologic examinations.

Whole-brain MRI analysis for exploring the GMV differences between the groups revealed that CM had a larger thalamic GMV compared to TD, although no association was found between this GMV and the RNFL. In the V1 ROI analysis, a larger right V1 GMV was associated with thinner macular RNFL for both eyes. The state in which these brain regions remain larger can be interpreted as a delay from the structural reorganization of the adolescent brain, remaining in an immature state. Taken together, these results suggest that maltreated children have atypical differences not only in the sensory cortex but also in the sensory organs, specifically thinning parts of the retinal layers. However, it is necessary to address the neurobiological mechanisms that cause these changes in CM and how the effects on the sensory organs or sensory cortex occur.

The macular RNFL constitutes the most superficial layer of the ten distinct retinal layers, where the axons of the ganglion cells in the deeper layers converge. A large cross-sectional study (*N* = 865) reported that thinner RNFL and GCL (ganglion cell layer with inner plexiform layer) are associated with lower childhood or adulthood cognitive functions, suggesting sensitivity to brain function^34^. Additionally, the RNFL thinning observed in CM in our study is consistent with the most recent study reporting a reduction in the number of ganglion cells in a mouse model of childhood neglect exposure^31^. Generally, RNFL thinning is observed in conditions such as glaucoma and aging^36^. However, type 2 diabetes, although not a direct ophthalmic disease, can cause diabetic retinopathy, leading to reduced retinal thickness due to neurodegeneration. The pathophysiology of type 2 diabetes results in ineffective insulin, an inability to metabolize glucose, and chronic excess blood glucose. Although our OCTA results did not detect any differences between the groups related to capillaries in the retinal periphery, the thinner RNFL in CM may partially support this idea. This suggests impairment of capillaries adjacent to the deep retinal layers, which may have resulted in RNFL thinning. Such thinning occurs in the absence of diabetic retinopathy progression, as reported in a longitudinal study^37^. In our study, CM participants had a history of prolonged maltreatment for 2–16 years. If their hypothalamic-pituitary-adrenocortical (HPA) axis response to stress had been maintained, leading to a consistent release of high blood cortisol and glucose levels over a long-term period, excessive chronic glucose exposure may have impaired peripheral retinal vessels in CM. As CM participants have been in a secure environment for several years, their capillaries may have recovered from any retinal periphery damage. Thus, atypicality of the surrounding vessels may not have been observed by OCTA, even though the retinal neurodegeneration that once occurred could have remained. This situation might be analogous to the persistence of atypical brain structures in adulthood brought on by childhood maltreatment. In addition, numerous reports of higher cortisol levels in CM^12,38^ may support our speculation. The promoter of the glucocorticoid receptor gene (*NR3C1*) in the hippocampus of adults maltreated during childhood was highly methylated, leading to a low expression of this receptor^39^. That is, in individuals who have been maltreated in childhood, although decades ago, lower *NR3C1* gene expression in the brain suggests vulnerability in the negative feedback system of the HPA axis. Many other studies have reported the association of DNA methylation of the *NR3C1* with childhood maltreatment, not in brain tissue, but in peripheral tissues such as blood and saliva^40–43^. Thus, epigenetic changes in the *NR3C1* gene resulting from chronic extreme stress in CM may cause a vulnerable HPA axis. We speculate that this led to chronic excess cortisol and glucose secretion, resulting in micro-level impairment of periretinal capillaries over a long time and subsequent thinning of the RNFL.

The atypicality observed in CM may have originated either in the retina or V1. Neuronal connections exhibit bilateral selection, wherein the disruption of connections from either side, due to cell death or other causes, results in cell death in the opposite direction^44^. Assuming that retinal cells are impaired at the micro-level, resulting in a reduction in the number of axons going to the central nervous system, as manifested in the thinning of the RNFL, it is likely that this negative consequence extends to the projection site—specifically, the lateral geniculate nucleus in the thalamus and V1—delaying maturation due to a reduction in the number of cells and their axons. Conversely, given the vulnerability of the limbic system, responsible for interpersonal relationships and emotional regulation in CM^45^, it cannot be ruled out that atypicality in V1 may have originated from such subcortical vulnerability and subsequently affected the retina.

No significant group differences were observed in the color tests and visual cognitive tasks, except for the error score of tritanopes in FMT100. Compared to other forms of colorblindness, tritanopia, associated with this score, is more likely to be caused by environmental factors such as traumatic brain injury, diabetes, and macular degeneration, as well as genetic factors. However, the differences detected in this study might not be substantial enough to be concluded. This test was significantly influenced by cognitive function^46^, so the results likely do not simply reflect a lack of color vision, although age and FSIQ were adjusted in the group comparison analysis. There were also no significant correlations between the error scores of tritanopes in FMT100 and RNFL thickness (right; *r* = −0.19, *P* = 0.22, left; *r* = −0.25, *P* = 0.11). However, a significant group difference was observed, suggesting that this effect needs to be examined in further research. Significant group differences were detected in the total score and scores for the six subscales (color discrimination, light/dark adaptation, depth perception, peripheral vision, visual search, and visual processing speed) of the VAQ, a self-assessment instrument, indicating that the TD group was more aware of problems related to their vision than the CM group. However, as there were no group differences in objective ophthalmologic examinations, including visual acuity and astigmatism, it is possible that the CM group did not adequately self-evaluate their vision. As a previous study has shown that childhood trauma, including maltreatment, affects metacognition, this result may also be related to the metacognitive problems of the children in the CM group^47^.

Group comparisons using whole-brain VBM revealed that the CM group had a larger thalamus than the TD group. This result aligns with initial findings in adolescents with RAD and young adults who witnessed domestic violence during childhood^6,48^. The thalamus is involved in the integration of perceptual, somatosensory, memory, and cognitive processes. Our finding that CM has a larger thalamic volume contradicts the findings of a study that adults who experienced childhood abuse had smaller thalamic GMV compared to healthy adults, and another study positing that higher scores on the Childhood Trauma Questionnaire (CTQ) were associated with smaller thalamic GMV in adult PTSD patients^49,50^. However, as adolescence is a transitional period with fluctuating brain volume, the direction of brain volume changes may differ from that in adults. Thus, the thalamus can be regarded as a brain region commonly affected by CM. Recent brain imaging studies on PTSD have consistently revealed a decrease in thalamic activity^51–54^. Given that eye movement desensitization and reprocessing (EMDR), which is clinically effective in trauma therapy, addresses sensory integration, repeated traumatic exposure to maltreatment may create vulnerabilities in the thalamus, which regulates sensory integration. Visual information is relayed through the lateral geniculate nucleus of the thalamus to V1. The influence of CM on this process is plausible, considering that relay synapses in the thalamus determine which signals to transmit to the brain. However, to date, a direct association between retinal thickness and thalamic GMV has not been demonstrated.

The GMV reduction in the left V1 observed in RAD (a more limited definition seen in CM but rarely observed) in our previous studies^4,15^ was not found in the whole-brain analysis in this population despite them having maltreatment experiences in common. In contrast, increased GMV in the right V1 was found in the ROI analysis conducted in this study, which seems to be a contradictory finding. This difference may have involved differences in developmental trajectories due to the mean age gap (almost two years), in addition to the presence of RAD diagnosis. Previous large studies in general adolescent populations have shown that cortical volume development follows an inverted U-shaped trajectory, peaking at eight to nine years^55^. A longitudinal study that examined this developmental trajectory in ASD and TD showed that the occipital lobe was thicker in ASD than in TD until age 16 and conversely became thinner in ASD than in TD from the late teens through adulthood^56^. In other words, children with neurodevelopmental disorders may exhibit atypicality in that their cortex becomes thicker than that of TD children after a certain period, or conversely, the direction of atypicality may change. The increased right V1 in CM observed in this study may also suggest atypical development, such as in a neurodevelopmental disorder. However, no longitudinal studies have revealed the brain volume developmental trajectory of CM, and it would be necessary to clarify the trajectory of brain development in CM in the future.

Although the present study reveals important findings, it has some limitations. First, it is not currently known whether examining retinal thickness can differentiate between other diseases and neurodevelopmental disorders, such as ASD. Several reports have confirmed that ASD and preterm infants also have atypical retinal thicknesses^57^; therefore, it is likely that this is not a feature found only in CM, and future research may provide an actual mechanistic explanation for the data provided in this study. However, the simultaneous presence of immature visual pathways, such as the larger thalamus and V1, and thinner RNFL may be unique to CM. Second, several underlying factors, such as prenatal tobacco or alcohol abuse, may contribute to both abusive experiences and retinal atypicality. It has been reported that exposure to tobacco smoke from in utero through childhood is associated with thinner RNFL in young adulthood^58,59^. Although the participants in this study were elementary to high school students, which is a different age group from the prior studies cited, we could not determine the effect of in utero exposure to cigarette smoke in the sample because of a lack of data on this aspect. Furthermore, the effects of fetal alcohol exposure on RNFL have not been consistently reported and will require further validation^59,60^. Therefore, determining the relevance of diverse background factors that may influence these outcomes may be necessary. Third, the segmentation of the retinal layers should have been considered in more detail. We used a simple stratification technique commonly used in clinical practice. Further insights may be gained by examining images with finer resolution. Finally, the color test and visual cognition task required a cognitive component that did not allow the experiment to be conducted solely on the visual component. Therefore, more primitive visual-sensory tasks should be performed.

In conclusion, examining retinal thickness in children could be a clinical indicator of immaturity in visual pathway development, as found in CM. We also need to clarify which phenotypic features might be involved in this reduced retinal thickness. It has been partially demonstrated that this might not be associated with a substantial loss of visual function, such as visual acuity. Therefore, it may be linked to behavioral difficulties, such as interpersonal relationships and emotional regulation, through effects on higher parts of the brain, such as V1 vulnerability**.**

## Methods

### Ethics statement

The Ethics Committee of the University of Fukui, Japan (approval nos. 20210004, 20200047, and 20220039), approved this study’s protocol. The study was conducted in accordance with the Declaration of Helsinki and the Ethical Guidelines for Clinical Studies of the Ministry of Health, Labour, and Welfare of Japan. Written informed consent from parents or the director of the child welfare facility was obtained.

### Participants

This study, a subset of a longitudinal study, involved 21 Japanese children and adolescents aged 9–18 years (12 boys, 9 girls; mean age ± SD; 14.0 ± 2.7 years) with CM experiences. Recruitment sources included the University of Fukui Hospital and local child welfare facilities (Table 1). All CM participants had experienced physical, emotional, and/or sexual abuse and neglect, leading to care by the Child protective service (CPS) or equivalent. Of the CM group, 20 children lived in stable environments within child welfare facilities, while one child lived with a non-perpetrator parent after receiving temporary shelter from CPS. Two children were diagnosed with neurodevelopmental disorders according to DSM-5 criteria; attention-deficit/hyperactivity disorder (ADHD) (1), and ASD with ADHD (1). Most CM participants (87.0%) were medication naïve, except for three (13.0%) who underwent a washout 72 h before scanning (methylphenidate, atomoxetine, and risperidone). The non-maltreated group consisted of 23 TD Japanese children (11 boys, 12 girls; mean age ± SD; 14.3 ± 2.6 years) with no maltreatment history, recruited from the local community via advertisements, and matched for age and sex. Exclusion criteria for the TD group included psychiatric diagnoses (mood-related disorders, anxiety disorders, and stress disorders) and neurodevelopmental disorders (ASD, ADHD, and learning disorders). For all participants, exclusion criteria involved a Full-Scale Intelligence Quotient (FSIQ) < 70 on the Wechsler Intelligence Scale for Children-Fourth Edition or the Wechsler Adult Intelligence Scale-Third Edition, histories of head trauma with loss of consciousness, perinatal or neonatal complications, neurological disorders, sleep disturbances, or medical conditions adversely affecting growth and development.

### Ophthalmologic examinations

All participants underwent comprehensive ophthalmic examinations, including best-corrected visual acuity (BCVA) testing, slit-lamp biomicroscopy, intraocular pressure measurement using Goldmann applanation tonometry (Luneau Technology Operations, Normandie, France), critical fusion frequency, Ishihara color blindness test (for screening color blindness), and dilated fundoscopy.

### Visual Activities Questionnaire (VAQ)^32^

The VAQ is a self-report questionnaire consisting of 33 items across eight subscales (color discrimination, glare disability, light/dark adaptation, acuity/spatial vision, depth perception, peripheral vision, visual search, and visual processing speed). Seven items (13, 14, 22, 24, 29, 30, and 31) pertaining to driving were excluded, in accordance with an ADHD study in children^61^. The average score for each subscale was used in the analysis.

### Farnsworth-Munsell 100-hue Test (FMT100)^33^

The FMT100 objectively assessed color discrimination ability under standard light conditions (D65 daylight, 6,500 K) in the same room and place. The test required participants to sequence color reference caps in the order of incremental hue variations spanning the visible spectrum. In addition to the total error score, error scores reflecting the number of misplacements were calculated separately for protanopes, deuteranopes, and tritanopes.

### Optical Coherence Tomography (OCT) and OCT Angiography (OCTA)

**Figure 3:**
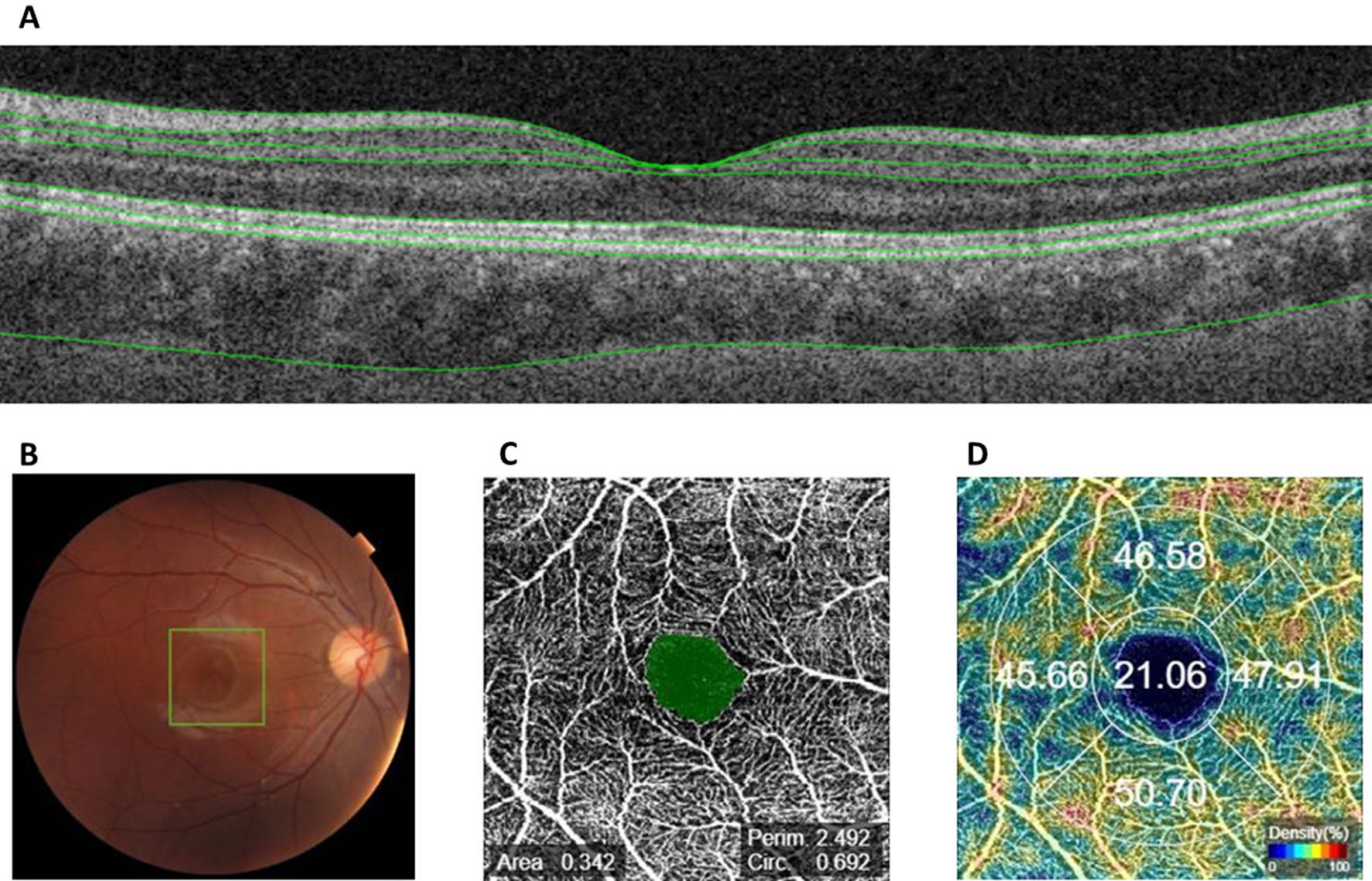
OCT, OCTA images. (**A**) OCT images automatically segmented at the Internal Limiting Membrane, RNFL/GCL, GCL/ Inner Plexiform Layer (IPL), IPL/Inner Nuclear Layer, photoreceptor inner segment junction/outer segment junction, Cone Outer Segment Termination, Retinal Pigment Epithelium, and choroidal scleral junction. (**B**) Fundus photograph taken simultaneously with OCTA. A 3 mm × 3 mm area centered on the fossa indicated by the green square was analyzed by OCTA. (**C**) FAZ (area, perimeter, circularity). (**D**) Vascular Density.

OCT and OCTA images, including central retinal thickness (CRT) measurements, were captured using Triton OCT (Topcon Medical Systems, Inc., Oakland, NJ, USA). Fluorescein Angiography (FA) images were captured using Spectralis Heidelberg Retinal angiography (Heidelberg Engineering, Heidelberg, Germany). All imaging tests were conducted by experienced orthoptists blinded to group status. OCT examined retinal thickness within a 6 mm × 6 mm area centered on the fovea, based on the Early Treatment Diabetic Retinopathy Study (ETDRS) grid, using the built-in automatic segmentation system. The defined segments included RNFL, GCL+, combined RNFL and GCL+ (GCL++), choroid (from the branch membrane to choroidal scleral junction), and retina (from the internal limiting membrane to the outer segment/retinal pigment layer). The average thickness of each segment was used for subsequent statistical analyses. OCTA analysis was conducted within a 3 mm × 3 mm area centered on the fovea. The built-in system automatically evaluated the area, perimeter, and circularity of the foveal avascular zone (FAZ) and vessel density in five regions (central, inferior, nasal, superior, and temporal) according to the ETDRS grid (Fig. 3). To prevent segmentation errors, an ophthalmologist (Y.Y) specializing in the retina reviewed OCT and OCTA images for all cases. Notably, no segmentation errors were identified in this study that could distort the results of statistical analyses.

### Visual cognitive tasks

The experiments were conducted in a quiet room at the university, with participants seated approximately 50 cm in front of a 19-inch monitor. Visual cognitive tasks were implemented and presented using the PsyToolkit platform^62,63^. All tasks were conducted using the default settings of PsyToolkit, with instructions translated into Japanese. For “Visual search^64,65^, participants were instructed to press the space button if they identified the letter “T” among 5–20 presented items, only if it was in its regular upright position and orange. No action was required if no “T” was present. This procedure was repeated for 50 trials with recording reaction times (RT) and error rates for each occurrence of the target “T” and when it was not present (non-target). For the “Navon task”^66,67^, participants were asked to press the B button if they saw the letter “H” or “O” and the N button if they saw neither an “H” nor “O.” The presentation of “H” and “O” was randomly assigned to emphasize either global or local features. In each of the 50 trials, participants had up to four seconds to determine whether they observed a target letter at the global or local level. The RT and error rates were recorded for each case (Global, Local, or Non-target). For the “Cueing, Posner Task”^68^, participants were requested to press the A button if they observed a go signal in the left box and the L button if they observed a go signal in the right box. After the stimulus was presented, a cue (indicated by the X mark) appeared over the left or right box, which may or may not anticipate the correct cue. The button on the side where the go signal appeared must be pressed to avoid being misled by it. There were 100 trials, and 75% of the cues represented valid directions, although not all trials had cues. The RT and error rates were recorded for each case (Valid or Invalid). In the “Mental rotation” task^69,70^, participants were asked to classify, using a mouse click, which figures presented at both the bottom left and right sides matched the two-dimensional stimulus figure presented on the top. The bottom figures were displayed in a rotated state, requiring participants to mentally rotate one or both figures. There were 10 trials, and the RT and error rates were recorded.

### Structural MRI acquisition

Images of 46 participants were acquired using a 3.0 Tesla General Electric SIGNA MRI system (Signa PET/MR, GE Healthcare, Milwaukee, WI, USA) equipped with an 8-channel head coil. A T1-weighted anatomical scan was obtained using a fast spoiled-gradient recalled imaging sequence with the following parameters; Repetition Time (TR) = 8.488 ms, Echo Time (TE) = 3.248 ms, Flip angle (FA) = 11°, Field of view (FOV) = 256 mm, matrix size = 256 × 256, volume dimensions = 1.0 × 1.0 × 1.0 mm^3^, slice thickness = 1.0 mm, and a total of 172 slices.

### Preprocessing of structural images for VBM analysis

The scan data, originally in the Digital Imaging and Communication in Medicine (DICOM) image format, were converted to the Neuroimaging Informatics Technology Initiative (NIfTI) image format using MRIcron software (https://www.nitrc.org/projects/mricron). Preprocessing and statistical analysis of the structural brain image data were conducted using the Statistical Parametric Mapping 12 software (SPM12) developed by the Wellcome Department of Cognitive Neurology in London, UK, and implemented using MATLAB (version 9.0; MathWorks Inc. Natick, MA, USA).

Appropriate pre-processing steps were applied to the children’s data. First, the T1-weighted structural images of each individual were segmented into gray matter, white matter, and cerebrospinal fluid using the segmentation algorithm available in SPM12. Second, the gray matter tissue probability map (TPM) used in the analysis was adjusted based on the model fit and tailored to the demographics of the specific pediatric population of interest. Third, the segmented gray matter and white matter tissues from all subjects were utilized to generate a customized template using Diffeomorphic Anatomical Registration through the Exponentiated Lie Algebra (DARTEL) algorithm. This step ensured accurate intersubject registration, particularly in improving the alignment of smaller inner structures. Default parameters were used during the segmentation process, except for affine regularization, which utilized the International Consortium for Brain Mapping (ICBM) template specifically designed for East Asian brains.

The resulting images were further normalized to the Montreal Neurological Institute (MNI) space through affine transformation, resulting in the creation of the DARTEL template. Subsequently, the segmented images of each subject were nonlinearly transformed to align them with the DARTEL template. Gaussian smoothing with a full width at half-maximum (FWHM) of 6mm was applied to gray matter images during the normalization process.

The imaging data were analyzed using SPM12 software. First, a whole-brain two-sample model was employed to explore potential differences in regional GMV changes between the CM and TD groups. The model included age, sex, FSIQ, dominant hand, and GMV as covariates, with the variances associated with these factors excluded from the analyses. Second, ROI analysis was conducted, specifically targeting the bilateral V1 as ROIs based on prior hypotheses of atypical V1 development, as observed in a previous study^4,15^. The ROIs were defined using the automated anatomical labeling (aal) method implemented in the WFU Pick-Atlas toolbox, Version 3.0.5^71–73^. Both whole-brain and ROI analyses were conducted, and corrections for multiple comparisons at the cluster level were applied to examine the GMV differences between groups. The statistical threshold was set at the voxel level *P* < 0.001 at the cluster level, with a family wise error (FWE) correction and a threshold set to *P* < 0.05. The Neuromorphometrics Atlas provided by SPM12 (Neuromorphometrics, Inc.; http://www.neuromorphometrics.com/) was used to determine the anatomical localization of the significant clusters.

### Statistical analysis

For group comparisons of clinical and psychological variables, Welch’s *t*-test and multiple linear regression analysis using heteroscedasticity-robust standard errors with age and FSIQ as covariates were used. Since the clinical and psychological assessments used in this study seemed to be influenced by cognitive function, we considered differences between groups significant if they were identified by multiple regression analysis. Pearson correlation analysis was conducted to investigate the potential association between retinal thickness and GMV. Additionally, Pearson correlation analysis using potential indices of maltreatment severity was conducted with retinal thickness. The analysis focused on the residuals obtained from group comparisons of the GMV, which were adjusted for control variables (β values). The adjusted eigenvariates, representing linearly transformed estimates of GMV, were used for correlation analysis. All statistical analyses were conducted using R software (version 4.2.1)^74^.

### Supplementary Information

Supplementary Tables.

## Data Availability

The original contributions presented in the study are included in the article and its supplementary material. Further inquiries can be directed to the corresponding authors.
