# Supplementary material for: Subclinical structural atypicality of retinal thickness and its association with gray matter volume in the visual cortex of maltreated children
Source: Sci Rep. 2024 May 20;14:11465. doi: 10.1038/s41598-024-62392-6 (PMC11106279; doi:10.1038/s41598-024-62392-6)
Supplement: Supplementary file 1 — Supplementary Tables. [file 41598_2024_62392_MOESM1_ESM.docx]

Supplementary Table S1. Visual Activities Questionnaire (VAQ)

|  | CM (*n* = 21) | TD (*n* = 23) | Welch *t*-test | | Multiple regression | |
| --- | --- | --- | --- | --- | --- | --- |
|  |  |  | *t* | *P* | *t* | *P* |
| Color discrimination | 1.9 ± 0.4 | 4.3 ± 0.5 | -3.91 | 0.0003**** | -3.50 | 0.001*** |
| Glare disability | 1.0 ± 0.2 | 1.9 ± 0.3 | -2.62 | 0.01* | -1.95 | 0.06 |
| Light/dark adaptation | 4.4 ± 0.7 | 9.5 ± 0.9 | -4.48 | 5.75e-05**** | -3.92 | 0.0003**** |
| Acuity/spatial vision | 3.6 ± 0.6 | 5.9 ± 0.5 | -2.79 | 0.008** | -1.90 | 0.07 |
| Depth perception | 1.8 ± 0.4 | 4.2 ± 0.4 | -4.25 | 0.0001**** | -3.63 | 0.0008**** |
| Peripheral vision | 2.9 ± 0.6 | 6.1 ± 0.5 | -4.25 | 0.0001**** | -3.83 | 0.0004**** |
| Visual search | 4.2 ± 0.7 | 9.1 ± 0.7 | -4.82 | 1.94e-05**** | -4.26 | 0.0001**** |
| Visual processing speed | 2.8 ± 0.5 | 5.2 ± 0.3 | -3.76 | 0.0006**** | -3.15 | 0.003*** |
| Total | 22.5 ± 3.6 | 46.2 ± 3.2 | -4.95 | 1.32e-05**** | -4.33 | 9.57e-05**** |

Data are shown as mean ± standard error. Multiple regression analyses were adjusted with age and full-scale IQ.

*; *P* < 0.05, **; *P* < 0.01, ***; *P* < 0.005, ****; *P* < 0.001

Supplementary Table S2. Farnsworth-Munsell® 100 Hue Test

|  | CM (*n* = 21) | TD (*n* = 21) | Welch *t*-test | | Multiple regression | |
| --- | --- | --- | --- | --- | --- | --- |
|  |  |  | *t* | *P* | *t* | *P* |
| Total error score (TES) | 87.4 ± 18.0 | 47.7 ± 7.4 | 2.04 | 0.05 | 1.44 | 0.16 |
| Protanopes | 12.3 ± 3.1 | 7.1 ± 1.7 | 1.49 | 0.15 | 0.87 | 0.39 |
| Deuternanopes | 9.3 ± 2.5 | 6.7 ± 1.5 | 0.90 | 0.38 | 0.19 | 0.85 |
| Tritanopes | 20.0 ± 3.0 | 11.4 ± 1.5 | 2.54 | 0.02* | 2.15 | 0.04* |

Data are shown as mean ± standard error. Multiple regression analyses were adjusted with age and full-scale IQ.

*; *P* < 0.05

Supplementary Table S3. OCTA

|  | CM (*n* = 21) | TD (*n* = 22) | Welch *t*-test | | Multiple regression | |
| --- | --- | --- | --- | --- | --- | --- |
|  |  |  | *t* | *P* | *t* | *P* |
| **OD (Right)** |  |  |  |  |  |  |
| Image quality | 75.6 ± 0.9 | 74.9 ± 1.1 | 0.50 | 0.62 | ﻿0.66 | 0.51 |
| FAZ |  |  |  |  |  |  |
| area | 0.3 ± 0.0 | 0.3 ± 0.0 | 1.21 | 0.23 | 1.50 | 0.14 |
| perimeter | 2.5 ± 0.1 | 2.4 ± 0.1 | 1.42 | 0.16 | 1.76 | 0.09 |
| circularity | 0.7 ± 0.0 | 0.7 ± 0.0 | -0.51 | 0.61 | -0.55 | 0.58 |
| Density map |  |  |  |  |  |  |
| C | 20.4 ± 2.1 | 20.2 ± 0.7 | 0.12 | 0.91 | 0.29 | 0.77 |
| I | 48.8 ± 0.6 | 49.0 ± 0.8 | -0.22 | 0.83 | 0.32 | 0.75 |
| N | 46.5 ± 0.5 | 46.5 ± 0.5 | 0.07 | 0.94 | 0.34 | 0.73 |
| S | 49.4 ± 0.7 | 49.1 ± 0.8 | 0.37 | 0.72 | 1.10 | 0.28 |
| T | 47.8 ± 0.6 | 47.7 ± 0.4 | 0.12 | 0.90 | 1.11 | 0.91 |
| **OS (Left)** |  |  |  |  |  |  |
| Image quality | 76.6 ± 1.0 | 76.4 ± 1.3 | 0.10 | 0.92 | ﻿0.79 | 0.43 |
| FAZ |  |  |  |  |  |  |
| area | 0.3 ± 0.0 | 0.3 ± 0.0 | 0.73 | 0.47 | 1.26 | 0.21 |
| perimeter | 2.4 ± 0.1 | 2.4 ± 0.1 | 0.09 | 0.93 | 0.56 | 0.58 |
| circularity | 0.7 ± 0.0 | 0.6 ± 0.0 | 1.71 | 0.10 | 1.59 | 0.12 |
| Density map |  |  |  |  |  |  |
| C | 19.8 ± 1.6 | 20.4 ± 0.9 | -0.31 | 0.76 | -0.63 | 0.53 |
| I | 49.2 ± 0.5 | 48.6 ± 0.7 | 0.76 | 0.45 | 0.87 | 0.39 |
| N | 48.2 ± 0.9 | 47.9 ± 0.4 | 0.28 | 0.78 | 0.85 | 0.40 |
| S | 49.8 ± 0.8 | 49.7 ± 0.5 | 0.18 | 0.85 | 1.15 | 0.26 |
| T | 48.2 ± 0.6 | 47.4 ± 0.4 | 1.00 | 0.33 | 1.86 | 0.07 |

Data are shown as mean ± standard error. Multiple regression analysis was adjusted with age and full-scale IQ.

FAZ; foveal avascular zone, C; central, I; inferior, N; nasal, S; superior, T; temporal

Supplementary Table S4. Visual cognitive tasks

|  | CM (*n* = 21) | TD (*n* = 23) | Welch *t*-test | | Multiple regression | |
| --- | --- | --- | --- | --- | --- | --- |
|  |  |  | *t* | *p* | *t* | *p* |
| Visual search |  |  |  |  |  |  |
| Non-target RT (msec) | 3921.6 ± 21.5 | 3965.9 ± 14.0 | -1.72 | 0.09 | ﻿-1.18 | 0.25 |
| Non-target error (%) | 3.1 ± 0.8 | 1.6 ± 0.7 | 1.42 | 0.16 | 0.93 | 0.36 |
| Target RT (msec) | 1332.7 ± 71.1 | 1084.1 ± 36.8 | 3.11 | 0.004*** | 1.55 | 0.13 |
| Target error (%) | 1.8 ± 0.8 | 0.9 ± 0.3 | 0.99 | 0.33 | 0.44 | 0.66 |
| Navon task |  |  |  |  |  |  |
| Global RT (msec) | 991.8 ± 36.7 | 888.4 ± 40.4 | 1.90 | 0.06 | 1.25 | 0.22 |
| Global error (%) | 17.1 ± 3.5 | 11.7 ± 2.3 | 1.29 | 0.21 | 1.46 | 0.15 |
| Local RT (msec) | 1010.5 ± 50.7 | 851.9 ± 28.7 | 2.72 | 0.01* | 1.65 | 0.11 |
| Local error (%) | 12.8 ± 2.6 | 11.4 ± 2.0 | 0.43 | 0.67 | 0.12 | 0.91 |
| Global/Local RT | 1.0 ± 0.0 | 1.0 ± 0.0 | -0.95 | 0.35 | -0.64 | 0.53 |
| Non-target RT (msec) | 1158.4 ± 59.5 | 970.8 ± 39.9 | 2.62 | 0.01* | 0.98 | 0.33 |
| Non-target error (%) | 6.7 ± 3.0 | 2.5 ± 0.9 | 1.30 | 0.20 | 1.10 | 0.28 |
| Mental rotation task |  |  |  |  |  |  |
| RT (msec) | 4313.3 ± 333.4 | 5033.7 ± 403.1 | -1.38 | 0.18 | -0.54 | 0.60 |
| error (%) | 32.9 ± 3.7 | 22.6 ± 2.5 | 2.30 | 0.03* | 0.16 | 0.87 |
| Posner task |  |  |  |  |  |  |
| Inv RT (msec) | 462.6 ± 22.9 | 437.0 ± 15.8 | 0.92 | 0.36 | -0.20 | 0.85 |
| Inv error (%) | 10.5 ± 3.0 | 7.6 ± 2.0 | 0.83 | 0.41 | 0.61 | 0.54 |
| Val RT (msec) | 411.0 ± 19.0 | 392.1 ± 15.0 | 0.78 | 0.44 | -0.07 | 0.94 |
| Val error (%) | 1.5 ± 0.6 | 0.5 ± 0.2 | 1.67 | 0.11 | 0.99 | 0.33 |
| Inv/Val RT (msec) | 1.1 ± 0.0 | 1.1 ± 0.0 | 0.22 | 0.83 | -0.56 | 0.58 |

Data are shown as mean ± standard error. Multiple regression analysis was adjusted with age and full-scale IQ.

*; *P* < 0.05, ***; *P* < 0.005
